# When flexibility is not necessarily a virtue: a review of hypermobility syndromes and chronic or recurrent musculoskeletal pain in children

**DOI:** 10.1186/s12969-015-0039-3

**Published:** 2015-10-06

**Authors:** Marco Cattalini, Raju Khubchandani, Rolando Cimaz

**Affiliations:** Pediatric Clinic, University of Brescia and Spedali Civili di Brescia, Brescia, Italy; Pediatric Rheumatology Clinic, Department of Paediatrics, Jaslok Hospital and Research Centre, Mumbai, India; Anna Meyer Children’s Hospital and University of Florence, Florence, Italy

**Keywords:** Hyperlaxity, Musculoskeletal pain, Ehlers-Danlos, Marfan, Loeys-Dietz, Stickler

## Abstract

Chronic or recurrent musculoskeletal pain is a common complaint in children. Among the most common causes for this problem are different conditions associated with hypermobility. Pediatricians and allied professionals should be well aware of the characteristics of the different syndromes associated with hypermobility and facilitate early recognition and appropriate management. In this review we provide information on Benign Joint Hypermobility Syndrome, Ehlers-Danlos Syndrome, Marfan Syndrome, Loeys-Dietz syndrome and Stickler syndrome, and discuss their characteristics and clinical management.

## Introduction

Chronic or recurrent musculoskeletal pain is a common complaint in children, affecting between 10 % and 20 % of children. It is one of the more frequent reasons for seeking a primary care physician’s evaluation and possible rheumatology referral [1, 2]. A wide variety of non-inflammatory conditions may cause musculoskeletal pain in the pediatric age, and the most common causes seen by paediatric rheumatologists include conditions associated with hypermobility. Hypermobility may have a significant impact on quality of life of affected children and their parents, even in the milder forms [3]. All physicians and allied professionals that may be involved in the care of children with musculoskeletal complaints should therefore be well trained to recognize hypermobility and to run the differential diagnosis between the various clinical entities associated with it. A critical approach to a child with hypermobility is crucial to correctly identify the underlying cause and avoid time/money- consuming investigations.

In this paper we review different etiologies of musculoskeletal pain associated with hypermobility, including some genetic disorders, and discuss the clinical approach in these children.

## Review

### Benign joint hypermobility syndrome (BJHS)

Children with hypermobile joints by definition display a range of movement that is considered excessive, taking into consideration the age, gender and ethnic background of the individual. It is estimated that at least 10–15 % of normal children have hypermobile joints and the term joint hypermobility syndrome (JHS) is reserved to the cases of joint hypermobility associated with symptoms with no other causes found for them [4–6]. JHS can be associated with hereditary connective tissue disorders, and the term “Benign” is used in contrast to more serious and potentially complicated or life-threatening musculoskeletal syndromes such as some forms of Ehlers-Danlos syndrome (EDS), Marfan syndrome, and Loeys-Dietz syndrome. The prevalence of JHS is not known with precision, given the lack of studies of large cohorts. Sperotto et al., conducted a cross sectional study in a cohort of healthy schoolchildren, aged 8–13 years from the province of Padua, Italy, and found that BJHS occurred in the 13,2 % of the 289 children evaluated [7].

Even if BJHS is very common, this condition is largely under-recognized by primary care physicians and often poorly managed. Symptoms frequently start in childhood and continue into adult life. The pathophysiology of benign joint hypermobility is unclear. Hypermobility is more common in childhood and adolescence, in females, and in some ethnicities, and it tends to lessen during adulthood. Still, polyarticular hypermobility may be present in up to 30 % of males and 40 % of females during early adulthood [8]. For the majority of individuals joint hypermobility may be of no consequence, and what brings a proportion of subjects to develop BJHS is not fully understood. BJHS seems to be transmitted by an autosomal pattern, and first-degree relatives with the disorders can be identified in many cases. Variable penetrance is generally observed [9]. With the exception of a minority of patients, who show a deficiency of tenascin X, no abnormality in collagen or related proteins has been identified as a cause for BJHS [10]. Joint pain is thought to be caused by excessive movement, increasing stress on joint surfaces, ligaments and adjacent structures. Other factors may contribute to the development of the syndrome, such as poor proprioception, autonomic dysfunctions and fatigue secondary to poor sleep [11].

The predominant presenting complaint is pain, which may be widespread and debilitating. The pain typically starts during or after activity. The most common affected sites are the lower limbs after walking (for example walking to and from school). Children usually report excess fatigue, handwriting difficulties or ‘clicking or cracking’ joints. Occasionally episodes of joint swelling lasting hours to days, joint dislocations, or more commonly subluxations with spontaneous reduction are reported. Back-pain is also a common complaint because the lumbar spine is one of the most mobile sections of the vertebral column and the excessive movements may lead to pain in hypermobile subjects. Heavy school bags are often an aggravating feature. Chronic pain results in a reduced exercise tolerance and can negatively impact patients’ life.

A significant proportion of subjects progressively quit sports and other physical activities. In addition, pain amplification is a common feature in these cases [12]. BJHS has been considered to cause only musculoskeletal symptoms for many years, but there is now mounting evidence that many other extra-skeletal manifestations may occur. This symptoms arise usually after the third decade of life, but have been described in adolescents, and may be due to connective tissue abnormalities, linking BJHS and other hereditary disorders of connective tissues, namely Ehlers-Danlos syndrome type III. These include functional and anatomic gastrointestinal tract abnormalities (constipation, bloating, diarrhea, hiatal hernias), autonomic dysfunctions (postural tachycardia syndrome, palpitations, orthostatic intolerance, headache, fatigue) and skin abnormalities (easy bruising, striae) [13, 14]. Some of these symptoms are overlapping with those observed in Juvenile Fibromyalgia (JFM), and indeed there are few reports describing high incidence of BJHS in children with JFM. Furthermore, children who have both JFM and BJHS may exhibit lower tender-points thresholds and a greater number of tender-points compared to children with JFM but no benign joint hypermobility [15].

**Fig. 1:**
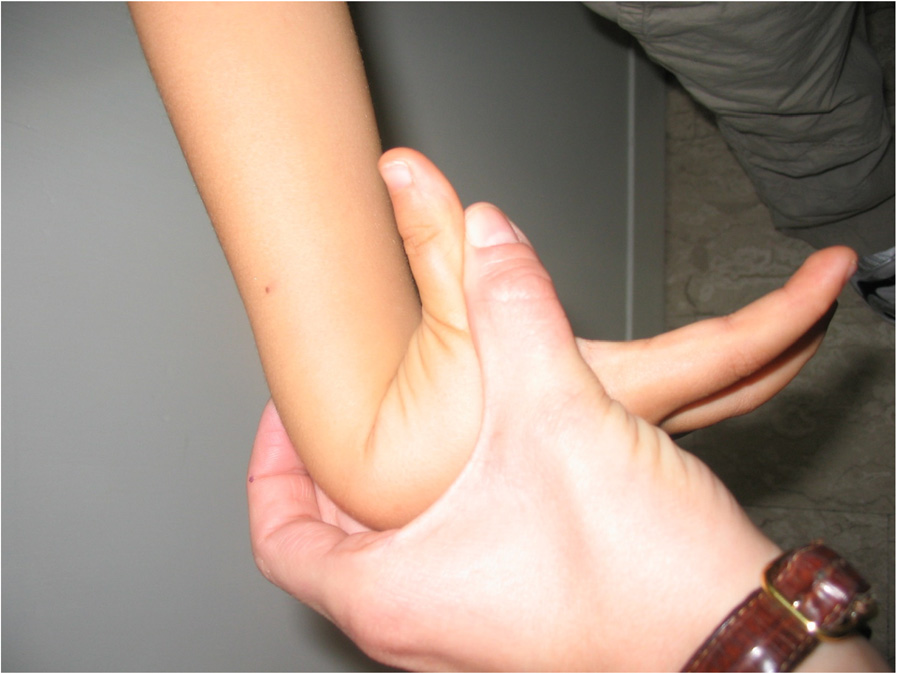
Passive apposition of thumb to the flexor aspect of forearm in a child with BJHS

**Fig. 2:**
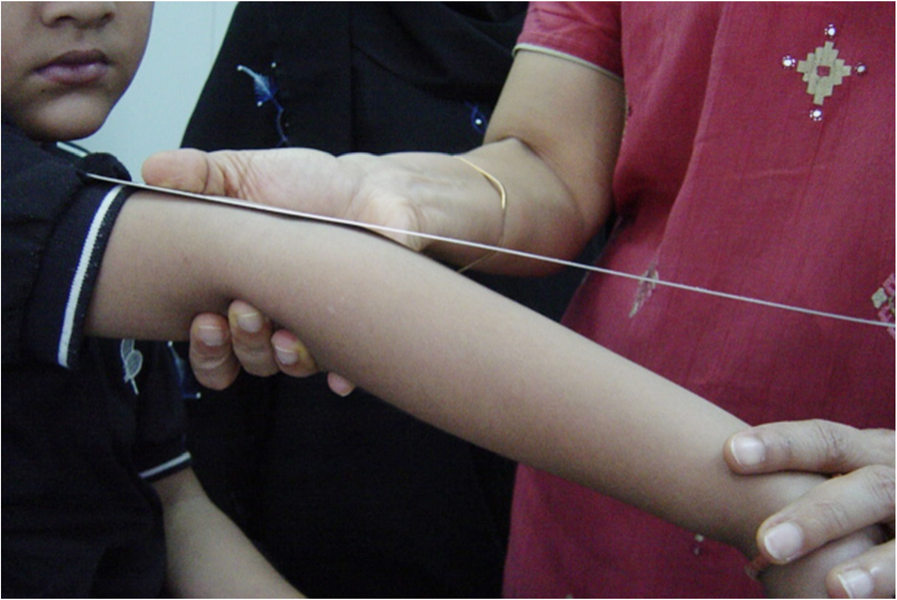
Hyperextension of the elbow in a child with BJHS

**Fig. 3:**
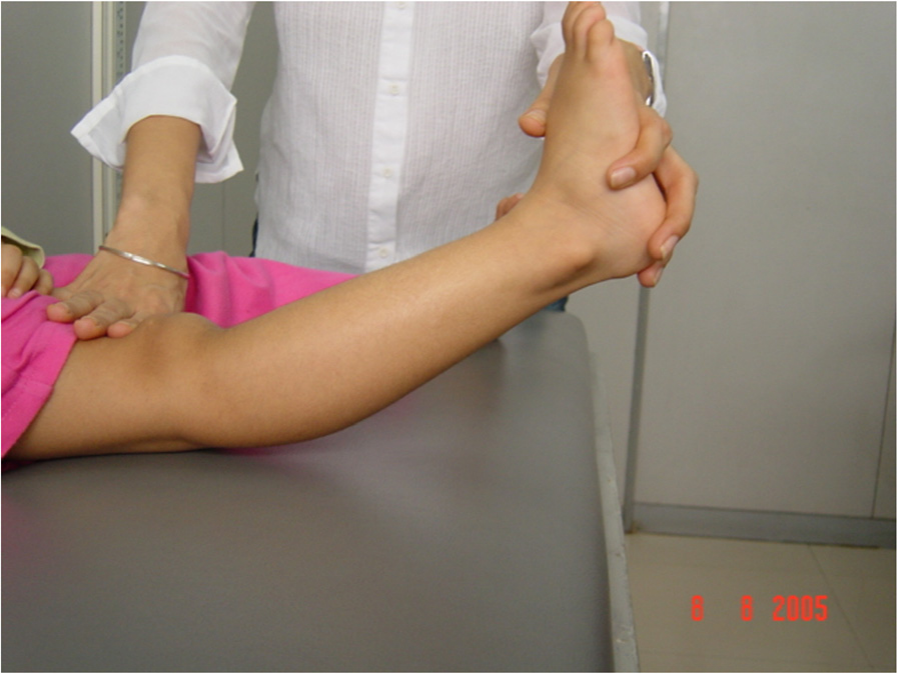
Hyperextension of the knee in a child with BJHS

The “Beighton score” (derived from the original one by Carter and Wilkinson) is commonly used to diagnose hypermobility. Hypermobility is present if 4 out of 9 points are scored. When this score is applied to normal children, a large proportion of the population is hypermobile (Table 1, Figs. 1, 2 and 3). This reflects the fact that, as already discussed, connective tissue structures may be looser and joints hypermobile in childhood, especially compared to adults. For these reasons it may be better to consider a Beighton score of 5 or more positive [16]. The Beighton score has been incorporated into a more comprehensive set of criteria called the Brighton Criteria (Table 2), which take into account the possible multisystemic nature of this condition. Although these criteria have not been formally validated in a pediatric population, they have been used in some studies on children with hypermobility [17–19].

**Table 1:** Beighton scoring system for joint hypermobility (adapted from Junge et al. [16])

Scoring 1 point each side
1. Passive dorsiflexion of the 5th metacarpophalangeal joint to >90°
2. Passive apposition of thumb to the flexor aspect of forearm
3. Hyperextension of the elbow >10°
4. Hyperextension of the knee >10°
Scoring 1 point
5. Flexion of the trunk with knees straight and both palms resting easily on floor

**Table 2:** The Brighton criteria for the diagnosis of BJHS (adapted from Graham et al. [17]

Major criteria
1. A Beighton score of 4/9 or greater (either currently or historically)
2. Arthralgia for longer than 3 months in four or more joints.
Minor criteria.
1. A Beighton score of 1,2, or 3/9 (0,1,2,or 3 if aged 50+).
2. Arthralgia (for 3 months or longer) in one to 3 joints or back pain for (for 3 months or longer), spondylosis, spondylolysis/spondylolisthesis.
3. Dislocation/subluxation in more than one joint, or in one joint on more than one occasion.
4. Soft tissue rheumatism: three or more lesions (e.g., epicondylitis, tenosynovitis, bursitis).
5. Marfanoid habitus (tall, slim, span/height ration >1.03 upper: lower segment ration less than 0.89, arachnodactyly (positive steinberg/wrist signs).
6. Abnormal skin striae, hyperextensibility, thin skin, papyraceous scarring.
7. Eye signs: drooping eyelids or myopia or antimongoloid slant.
8. Varicose veins or hernia or uterine/rectal proplapse.
BJHS is diagnosed in the presence of two major criteria or one major and two minor criteria, or four minor criteria. Two minor criteria will suffice where there is an unequivocally affected first-degree relative.

The management of individuals with BJHS can be very challenging and there are no evidence-based management strategies currently available. Acute pain episodes are commonly managed using taping, bracing or splinting or with non-steroidal anti-inflammatory drugs as needed. However reassurance and a multi-disciplinary training program are the mainstays of long term management. Physical therapy is of the outmost importance, and encouraging an active lifestyle may improve function and enhance quality of life [20]. As general principles, strengthening exercises focused on muscles around hypermobile joints may help to enhance joint support throughout movement and reduce pain; closed chain exercises may enhance proprioceptive feedback and optimize muscle action. Proprioception may be improved also by coordination and balance exercises. Physical therapy should also encompass a generalized exercise programme, addressing cardio-respiratory, musculoskeletal and neurological aspects of movement with the aim to reduce deconditioning [20]. As already mentioned, the Health Related Quality of Life in children with hypermobility and their parents may be worse than that of healthy controls and this may well be secondary to the presence of chronic pain and fatigue, that act as stressors in everyday life. This is an important aspect to consider for physicians approaching a child with BJHS, that should not underestimate the burden of this condition, often interpreted as benign and not worth any intervention [3].

### Ehlers-Danlos Syndromes (EDSs)

**Fig. 4:**
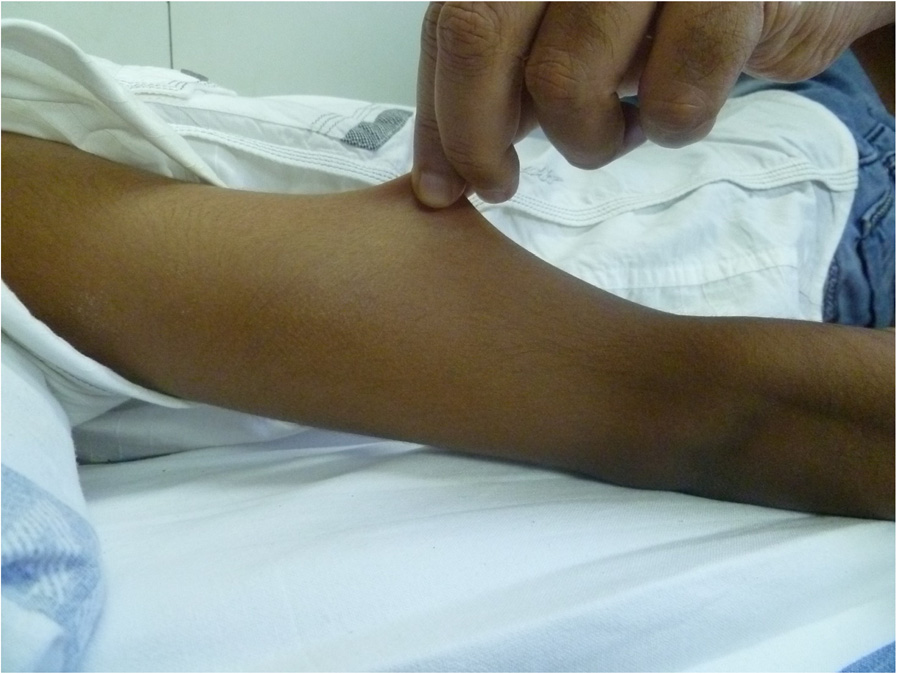
Skin hyper extensibility in a child with EDS subtype hypermobile

EDSs comprise a very heterogeneous group of heritable disorders of connective tissue. The increased flexibility and fragility of the soft connective tissues result in a wide range of changes in the skin, ligaments, joints, blood vessels and internal organs (Fig. 4). The current Villefranche classification recognizes six subtypes, according to clinical features, inheritance pattern and underlying molecular defects (Table 3). This classification underlines the extreme heterogeneity at both the clinical and the molecular level [21]. In the last few years, rarer phenotypes that do not fit in the Villefranche classification have been described. The prevalence of EDS is estimated to be approximately one in 5000 births, with no racial predisposition [22].

**Table 3:** Ehlers-Danlos Syndromes classification (adapted from Beighton P et al. [21])

Type	Clinical manifestations		IP^a^	Protein	Gene
	Major criteria	Minor criteria			
Classic (type I/II)	Skin hyperextensibility	Easy bruising	AD	Type V procollagen (~50 %)	*COL5A1*
	Widened atrophic scarring	Molluscoid pseudotumors			*COL5A2*
	Joint hypermobility	Subcutaneous spheroids			
	Smooth and velvety skin	Muscular hypotonia			
		Complications of joint hypermobility			
		Surgical complications			
		Positive family history			
Hypermobility (type III)	Generalized joint hypermobility mild skin involvement	Recurring joint dislocations	AD	Tenascin X (~5 %)	*TNX-B*
		Chronic joint pain			
		Positive family history			
Vascular (type IV)	Excessive bruising	Acrogeria	AD	Type III procollagen	*COL3A1*
	Thin, translucent skin	Early-onset varicose veins			
	Arterial/intestinal/uterine fragility or rupture	Hypermobility of small joints			
	Characteristic facial appearance	Tendon and muscle rupture			
		Arteriovenous or carotid-cavernous sinus fistula			
		Pneumo (hemo)thorax			
		Positive family history, sudden death in close relative(s)			
Kyphoscoliotic (type VI)	Severe muscular hypotonia at birth	Tissue fragility, including atrophic scars	AR	Type VIA: Lysyl hydroxylase 1	*LH-1 (PLOD1)*
	Generalized joint laxity	Easy bruising			
	Kyphoscoliosis at birth	Arterial rupture			
	Scleral fragility and rupture of the globe	Marfanoid habitus		Type VIB: not known	
		Microcornea			
		Osteopenia			
Arthrochalasis (type VII A & B)	Severe generalized joint hypermobility with recurrent subluxations	Skin hyperextensibility	AD	Type I procollagen	*COL1A1*
	Congenital bilateral hip dislocation	Tissue fragility, including atrophic scars			*COL1A2*
		Easy bruising			
		Muscular hypotonia			
		Kyphoscoliois			
		Mild osteopenia			
Dermatosparaxis (type VII C)	Severe skin fragility	Soft, doughy skin texture		Procollagen-N-proteinase	*ADAMTS-*2
	Sagging, redundant skin	Premature rupture of membranes			
	Excessive bruising	Large herniae			

^a^ *IP* inheritance pattern

*AD* Autosomal Dominant, *AR* Autosomal Recessive

The connective tissue fragility follows abnormalities in the expression or structure of the fibrillar collagen types I, III and V, as well as enzymatic abnormalities in the post-translational modification and processing of these collagens. Mutations in the COL5A1 and COL5A2 genes, encoding the α1- and the α2-chains of type V collagen, respectively, are found in approximately 50 % of individuals with the classic type of EDS. Mutations in TNX-B, encoding for Tenascin X, in approximately 5 % of patients with the hypermobility type, while vascular EDS is caused by heterozygous mutations in the COL3A1 gene, encoding type III collagen [23, 24].

The clinical spectrum of EDSs varies from mild skin and joint hyperlaxity to severe physical disability and life-threatening vascular complications. The classic, hypermobility and vascular subtype of EDS are the most common, whereas the kyphoscoliosis, arthrochalasis and dermatosparaxis types are very rare conditions. The diagnosis of the autosomal dominant (AD) classic subtype of EDS requires the presence of skin hyper-extensibility, widened atrophic scars and joint hypermobility. These are the three major diagnostic criteria, next to a series of ‘minor’ diagnostic manifestations. Characteristic facial features include epicanthic folds, excess skin over the eyelids, presence of dilated scars on the forehead and vaulted palate. Joint hypermobility is usually generalized and can vary in severity and with age. At birth, uni- or bilateral dislocation of the hip may be present. Even if the hypermobility is asymptomatic, this condition can result in childhood in congenital club foot, pes planus and joint effusions. In young adulthood the classic subtype can be complicated by repetitive subluxations and dislocations either spontaneously or after minimal trauma. Patients usually report chronic and recurrent pain that is difficult to treat and premature osteoarthritis is a major concern. One of the most typical features is the skin hyper-extensibility, which means that the skin stretches easily but snaps back after release. The skin is often smooth and velvety to the touch [23] (Fig. 4).

For pediatric rheumatologists, a real diagnostic challenge is represented by the hypermobility subtype of EDS (EDS-HT), which is by far the most common subtype. The genetic basis of EDS-Hybermobile is largely unknown and a reliable diagnostic test for this EDS subtype is lacking [25]. According to the Villefranche classification, the major diagnostic criteria are generalized joint hypermobility and presence of typical skin manifestations. However, these features are usually more subtle than in the classic type of EDS but these criteria are nevertheless helpful to differentiate this form of EDS from the more common “Benign joint hypermobility syndrome (BJHS)” [26]. It is still a matter of debate if EDS-HT and BJHS really represents two different diseases or if they should be reviewed as a spectrum of a single entity, sharing common genetic bases and showing considerable variability in clinical presentation, between as well as within families.

Joint hypermobility is typically limited to the small joints of the hands in the vascular subtype. This subtype has the worst prognosis because of a high rate of spontaneous arterial rupture usually in the third or the fourth decade of life. Unlike other EDS types, the skin is not hyper-extensible, but rather thin and translucent, showing a visible venous pattern over the chest, abdomen and extremities. Excessive bruising is the most common sign and is often the presenting complaint, especially in children. Other early manifestations include premature rupture of the membranes, congenital clubfoot or congenital hip dislocation, inguinal hernia, and severe varicosities. The facial and cutaneous features may be very subtle or even absent [27]. If there is a strong clinical suspicion of vascular EDS, direct DNA analysis is mandatory, even in the absence of an abnormal biochemical abnormality.

The management of children with Ehlers-Danlos syndromes requires a multidisciplinary approach. Children with pronounced skin fragility should be advised to avoid contact sports and to wear protective pads or bandages in order to prevent bruises and hematomas. Cutaneous stitches should be left in place twice as long as usual, and additional fixation of adjacent skin with adhesive tape can help to prevent stretching of the scar. In children physio-therapeutic support is important. Acetaminophen should be preferred over NSAIDs for joint pain and thus minimizing the risk of easy bruising due to platelet disfunction. For the same reason COX-2 inhibitors may be an option, although no studies have been published on their use in EDS. Patients with mitral valve prolapse and regurgitation require antibiotic prophylaxis for bacterial endocarditis. A baseline echocardiogram with aortic diameters measurement is recommended before 10 years of age, with follow-up studies timed according to whether an abnormal measurement is found. A useful resource for these measurements is *parameterz.blogspot.in*. For the vascular and vascular-like types of EDS, some prophylactic measures are of particular importance. Invasive vascular procedures such as arteriography and catheterization should also be avoided because of the risk for life-threatening vascular rupture. Surgical interventions are generally discouraged because of increased vascular fragility, and conservative therapy is recommended [28, 29].

### Marfan Syndrome (MS)

MS is an hereditary autosomal dominant, multisystem disorder of connective tissue with extensive clinical variability. It is a relatively common condition, with approximately 1 in 5000 people affected. This disease demonstrates autosomal-dominant inheritance with high penetrance and marked inter- and intra-familial variability [30]. It is caused by defects in FBN1, the gene that codes for the protein fibrillin, although patients with mutations in other genes, including TGFBR1 and TGFBR2, have also been rarely reported [31]. Mutations in FBN1 are associated with a wide phenotypic spectrum ranging from classic features of Marfan syndrome presenting in childhood and early adulthood to severe neonatal presentation [32]. Advanced paternal age is a risk factor.

Cardinal features involve the ocular, musculoskeletal, and cardiovascular systems. Skeletal system involvement in Marfan syndrome is characterized by bony overgrowth; such overgrowth may be noticeable at birth or can develop in young children and results in disproportional long limbs. Frequent findings are *pectus excavatum*, *pectus carinatum*, scoliosis or spondylolisthesis, calcaneal displacement, “*protrusio acetabuli*”, arachnodactyly, and *pes planus*. Recently, Marfan patients have been reported to have reduced bone mass and muscle mass, compared to healthy controls. All this skeletal abnormalities may account for the very high incidence of severe daily pain that Marfan patients report [33].

Joint laxity may be significant in young MS patients and can lead to ligament injury, dislocations, chronic joint pain and degenerative arthritis [34]. The facial features of Marfan syndrome include a long and narrow face with deeply set eyes (enophthalmos), downward slanting of the eyes, flat cheek bones (malar hypoplasia) and high arched palate [35]. *Ectopia lentis* (i.e., lens dislocation) is a cardinal feature of Marfan syndrome and an ophthalmologic examination is mandatory in suspected cases. During early childhood, patients may occasionally present with isolated bilateral *ectopia lentis* [36]. The cardiovascular involvement is particularly worrisome because the progressive aortic-root dilatation can lead to acute dissection, aneyrysms and sudden death. Although early diagnosis and refined medical and surgical treatment have improved survival, patients with Marfan syndrome continue to have high rates of cardiovascular disease and premature death [37].

The diagnosis is clinical, according to the revised Ghent criteria (Tables 4 and 5). These criteria however perform well in patients showing the typical phenotypes. These criteria are not as useful in milder MS variants, when only isolated features are present [38]. In young children Marfan syndrome is not always recognizable, especially in the absence of a family history, because many of the more specific clinical features are age dependent (e.g., *ectopia lentis*, aortic dilation, dural ectasia, *protrusio acetabuli*). A high index of suspicion is needed, and for subjects suspected to have Marfan syndrome based on clinical grounds FBN1 testing should be considered. A useful tool for risk stratification of suspected pediatric patients can be represented by the Kid-Short Marfan Score (Table 6) [39].

**Table 4:** The revised Ghent criteria for Marfan syndrome (adapted from Faivre L et al. [38])

In the absence of family history:
1. Aortic Root Dilatation Z score ≥ 2 AND Ectopia Lentis = Marfan syndrome.
2. Aortic Root Dilatation Z score ≥ 2 AND FBN1 mutations = Marfan syndrome.
3. Aortic Root Dilatation Z score ≥ 2 AND Systemic Score ≥ 7pts = Marfan syndrome.
4. Ectopia lentis AND FBN1 with known Aortic Root Dilatation = Marfan syndrome.
In the presence of family history:
1. Ectopia lentis AND Family History of Marfan syndrome (as defined above) = Marfan syndrome.
2. A systemic score ≥ 7 points AND Family History of Marfan syndrome (as defined above) = Marfan syndrome
3. Aortic Root Dilatation Z score ≥ 2 above 20 yrs. old, ≥ 3 below 20 yrs. old) + Family History of Marfan syndrome (as defined above) = Marfan syndrome.

**Table 5:** Revised Ghent criteria: systemic feature score (adapted from Faivre L et al. [38])

Table 2: Revised Ghent Criteria: Systemic features score
• Wrist AND thumb sign – **3** (Wrist OR thumb sign – **1**)
• Pectus carinatum deformity – **2** (pectus excavatum or chest asymmetry – **1**)
• Hindfoot deformity – **2** (plain pes planus – **1**)
• Pneumothorax – **2**
• Dural ectasia – **2**
• Protrusio acetabuli – **2**
• Reduced US/LS AND increased arm/height AND no severe scoliosis – **1**
• Scoliosis or thoracolumbar kyphosis– **1**
• Reduced elbow extension – **1**
• Facial features (3/5) – **1** (dolichocephaly, enophtalmos, downslanting palpebral fissures, malar hyoplasia, retrognathia)
• Skin striae – **1**
• Mitral valve prolapse (all types) – **1**
• Myopia *>*3 diopters – **1**
Maximum total: 20 points; score ≥7 indicates systemic involvement

Bold numbers refer to the points to be scored for each finding

**Table 6:** The Kids-Short Marfan score (adapted from Mueller GC et al. [39])

Required manifestations	Risk category for likelihood of MFS
Dilatation of aortic root + Ectopia lentis	Very high risk
• dilatation of aortic root	High risk (Complete examination of all symptoms of the revised Ghent Criteria is strictly recommended as soon as possible. Patient should see Marfan Syndrome specialists)
+ mitral valve prolapse + tricuspid valve prolapse or	
+ dilatation of pulmonary artery or	
+ at least 3 skeletal features of the systemic score of the revised Ghent criteria	
• Ectopia lentis	
+ mitral valve prolapse + tricuspid valve prolapse or	
+ dilatation of pulmonary artery	
Family history dilatation of aortic root	Moderate risk (Patient needs to be verified or excluded with further diagnostic procedures other than or echocardiography and clinical examination)

Diagnosis and management require a multidisciplinary approach by geneticists, cardiologists, orthopedic surgeons and ophthalmologists with experience in this field. Cardiovascular follow-up should include serial evaluation with electrocardiography and serial cardiac imaging, especially CT/MRI angiography. Exercise restriction is wise and elective aortic-root replacement is sometimes needed [40]. Infective endocarditis prophylaxis is indicated in those with valvular defects. Medical therapy with beta-blockers seems to be able to decrease aortic root enlargement, especially when started relatively early in the disease course, while the role of ACE inhibitors is still debated [41]. Readers are referred to *marfan.org*, an excellent resource for both physicians and patients

### Loeys-Dietz syndrome (LDS)

LDS is a recently described rare autosomal dominant connective tissue disorder characterized by a severe and widespread arterial involvement since childhood. Its exact incidence have not been established [42, 43]. The disorder is most often caused by heterozygous mutations in TGF-β receptors TGFBR1 and TGFBR2 [44]. The classification depends on the presence or absence of craniofacial features (hypertelorism, bifid uvula and cleft palate). Affected individuals show generalized arterial abnormalities, ascending aneurysms and rapidly progressive aortic aneurysm [45]. Skeletal features in all types of LDS may show overlap with Marfan syndrome (i.e., pectus deformity, arachnodactyly, scoliosis, and *pes planus*) but height and proportions are typically within the normal range. Joint hypermobility is also common. A more specific finding is the association of arachnodactyly with advanced carpal bone ossification and joint hyperextension [46, 47]. LDS diagnostic criteria have not been defined and confirmatory genetic testing is required. Management of LDS involves regular cardiology follow-up to establish the extent of vascular involvement, early surgical intervention, genetic counseling and monitoring in pregnancy. There is a higher risk of dissection compared to Marfan syndrome, and early surgical correction may be crucial. Aggressive medication regimens, with β-blockers and angiotensin receptor antagonists, is recommended as this treatment may halt disease progression and postpone surgical repair [48].

### Stickler syndrome (SS)

SS is a multisystem connective tissue disorder that can affect the eye, craniofacies, inner ear, skeleton, and joints [49]. Stickler syndrome has been associated with mutations of COL2A1, which encodes for the alpha-1 chain of type II collagen, COL11A1 gene, which encodes for the alpha-1 chain of type XI collagen, and COLL11A2 gene. Rarer autosomal recessive forms have been linked to mutation of the three genes encoding collagen IX: COL9A1-2-3. Variable phenotypic expression of Stickler syndrome occurs both within and among families [50–53].

Based on the vitreous abnormalities Stickler syndrome is classified as Type 1 (“membranous”; characterized by a persistence of vestigial vitreous gel in the retrolental space) and the rare Type 2 (“beaded”, characterized by sparse and irregularly thickened bundles throughout the vitreous cavity). A non-progressive myopia is common. Craniofacial findings may include a flat facial profile, telecanthus and epicanthal folds, micrognathia and cleft palate. Hearing impairment, especially sensorineural deafness for high tones, is common but the overall sensorineural hearing loss in type I Stickler syndrome is typically mild and not significantly progressive. The musculoskeletal features are early-onset arthropathy, short stature and mild spondyloepiphyseal dysplasia. In children and adolescents joint hypermobility is seen and usually becomes less prominent with age [52]. The diagnosis of Stickler syndrome is clinically based. At present, clinical diagnostic criteria have been proposed only for type 1 Stickler syndrome. Type 1 individuals have the membranous type of vitreous abnormality (see Table 7). The diagnosis of Stickler syndrome requires genetic analysis of the involved genes. The COL2A1 gene may be tested first in individuals with type 1 “membranous” vitreous abnormalities and milder hearing loss. COL1A1 mutations can be frequently found in patients with craniofacial and joint manifestations as well as hearing loss but without ocular findings [53].

**Table 7:** Proposed diagnostic criteria for Stickler Syndrome type I (adapted from Robin NH et al. [52])

Stickler syndrome should be considered in individuals with ≥5 points At least one finding should be a major (2-point) manifestation.
Abnormalities (2-pt maximum per category)
• Orofacial
○ Cleft palate* (open cleft, submucous cleft, or bifid uvula): 2 points
○ Characteristic facial features (malar hypoplasia, broad or flat nasal bridge, and micro/retrognathia): 1 point
• Ocular. Characteristic vitreous changes or retinal abnormalities* (lattice degeneration, retinal hole, retinal detachment or retinal tear): 2 points
• Auditory
○ High-frequency sensorineural hearing loss*: 2 points
▪ Age < 20 years: threshold ≥ 20 dB at 4–8 Hz
▪ Age 20–40 years: threshold ≥ 30 dB at 4–8 Hz
▪ Age > 40 years: threshold ≥ 40 dB at 4–8 Hz
○ Hypermobile tympanic membranes: 1 point
• Skeletal
○ Femoral head failure (slipped epiphysis or Legg-Perthes-like disease): 1 point
○ Radiographically demonstrated osteoarthritis before age 40: 1 point
○ Scoliosis, spondylolisthesis, or Scheuermann-like kyphotic deformity: 1 point
Family history/molecular data**
• Independently affected first-degree relative in a pattern consistent with autosomal dominant inheritance or presence of a *COL2A1*, *COL11A1*, or *COL11A2* pathogenic variant associated with Stickler syndrome**: 1 point
• *Denotes major manifestation ** Does not account for families with autosomal recessive Stickler syndrome

## Conclusions

Musculoskeletal pain is one of the more common complaints in the pediatric population. It is common for parents of affected children to seek a pediatric evaluation, and commonly these children are referred to a rheumatologist. Among the leading non-inflammatory causes of joint pain seen by pediatric rheumatologists are those associated with hypermobility. Different diseases are associated with hypermobility. The musculoskeletal complaints are typical of non-inflammatory conditions, with the onset of pain that is typically exacerbated by exercise and is usually a late afternoon/evening pain, without the typical signs and symptoms of inflammatory arthropathies, such as joint swelling or morning stiffness. Inflammatory markers are invariably within normal limits. Many other clinical manifestations may enrich the clinical picture, based on the underlying cause. The presence of chronic pain secondary to hypermobility may have a profound impact on patients’ quality of life, leading to a high burden for patients, families and the health care system. Moreover, the genetic syndromes depicted in this review may have early and late severe complications. Pediatricians, and specifically pediatric rheumatologists, should therefore keep a high index of suspicion for early recognition of hypermobility-associated syndromes. It is essential that these physicians have expertise in developing an appropriate differential diagnosis in these children and distinguishing one musculoskeletal disease from another.

## Consent

Written informed consent was obtained from the patient’s guardian/parent/next of kin for the publication of this report and any accompanying images.
